# TRAF2 Facilitates Vaccinia Virus Replication by Promoting Rapid Virus Entry

**DOI:** 10.1128/JVI.03013-13

**Published:** 2014-04

**Authors:** Ismar R. Haga, Tali Pechenick Jowers, Samantha J. Griffiths, Juergen Haas, Philippa M. Beard

**Affiliations:** a The Roslin Institute and Royal (Dick) School of Veterinary Studies, University of Edinburgh, Edinburgh, United Kingdom; b Division of Pathway Medicine and Centre of Infectious Diseases, University of Edinburgh, Edinburgh, United Kingdom

## Abstract

Tumor necrosis factor receptor (TNFR)-associated factor 2 (TRAF2) is a pivotal intracellular mediator of signaling pathways downstream of TNFR1 and -2 with known pro- and antiviral effects. We investigated its role in the replication of the prototype poxvirus vaccinia virus (VACV). Loss of TRAF2 expression, either through small interfering RNA treatment of HeLa cells or through genetic knockout in murine embryonic fibroblasts (MEFs), led to significant reductions in VACV growth following low-multiplicity infection. In single-cycle infections, there was delayed production of both early and late VACV proteins as well as accelerated virus-induced alterations to cell morphology, indicating that TRAF2 influences early stages of virus replication. Consistent with an early role, uncoating assays showed normal virus attachment but delayed virus entry in the absence of TRAF2. Although alterations to c-Jun N-terminal kinase (JNK) signaling were apparent in VACV-infected TRAF2^−/−^ MEFs, treatment of wild-type cells with a JNK inhibitor did not affect virus entry. Instead, treatment with an inhibitor of endosomal acidification greatly reduced virus entry into TRAF2^−/−^ MEFs, suggesting that VACV is reliant on the endosomal route of entry in the absence of TRAF2. Thus, TRAF2 is a proviral factor for VACV that plays a role in promoting efficient viral entry, most likely via the plasma membrane.

**IMPORTANCE** Tumor necrosis factor receptor-associated factors (TRAFs) are key facilitators of intracellular signaling with roles in innate and adaptive immunity and stress responses. We have discovered that TRAF2 is a proviral factor in vaccinia virus replication in both HeLa cells and mouse embryonic fibroblasts and that its influence is exercised through promotion of efficient virus entry.

## INTRODUCTION

Tumor necrosis factor receptor (TNFR)-associated factors (TRAFs) are key facilitators of intracellular signaling with roles in innate and adaptive immunity, stress responses, and bone metabolism (1). There are seven members of the TRAF family, TRAF1 to TRAF7, with all TRAFs except TRAF4 being involved in signaling downstream of the TNFR superfamily. TRAF2 mediates signaling of multiple pathways downstream of TNFR1 and -2, leading to the activation of both canonical and noncanonical NF-κB pathways (2), inhibition of apoptosis via interaction with caspase 8 (3, 4), and activation of the mitogen-activated protein kinases (MAPKs) p38 (5) and c-Jun N-terminal kinase (JNK) (6, 7).

JNK is a stress-activated protein kinase (SAPK) that is activated by cytokines, such as tumor necrosis factor alpha (TNF-α), by environmental stress, and also by intracellular stimuli, such as endoplasmic reticulum stress (8). The JNK signaling cascade includes various members of the MAPK kinase kinase (MAP3K) family which activate the MAPK kinases MKK4 and -7, leading to phosphorylation and activation of JNK. JNK substrates include not only nuclear transcription factors, such as AP-1 and c-Jun, but also nonnuclear proteins, such as the E3 ligase Itch; the mitochondrial antiapoptotic proteins Bcl2 and Bcl-xL; and regulators of cell movement, such as paxillin and microtubule-associated proteins MAP2 and MAP1B (8). These interactions allow the JNK pathway to influence a wide range of cell processes, including apoptosis, inflammation, protein degradation, cell cycle progression, and cytoskeletal regulation (9). Due to the multiple cellular functions regulated by JNK, manipulation of this signaling pathway is a strategy employed by a number of viruses, including poxviruses, in order to regulate cellular gene expression (10–14).

Poxviruses belong to the Poxviridae, a family of large DNA viruses that replicate entirely within the cytoplasm of the cell (15). The best characterized member of the family is Vaccinia virus (VACV), a member of the Orthopoxvirus genus, which also includes Cowpox virus, Ectromelia virus (the causative agent of mousepox), Monkeypox virus, and Variola virus, the causative agent of smallpox. VACV produces two forms of infectious progeny known as intracellular mature virus (IMV) and extracellular enveloped virus (EEV). IMVs consist of a core particle containing the viral genome and a selection of host and viral proteins surrounded by a single membrane. IMVs make up the majority of infectious progeny, and most remain inside the infected cell until cell lysis (16). However, a small proportion of IMVs are processed further and transported to the cell surface for release as double-enveloped EEV particles. EEVs represent only a minor proportion of the progeny virus in tissue culture, but they are biologically important, as they mediate the dissemination of virus within the infected host.

Once an EEV particle contacts an uninfected cell, it loses its outer membrane by nonfusogenic disruption (17), allowing the underlying IMV to enter the cell as a normal IMV would. Initially, the VACV IMV particle uses various host molecules, including glycosaminoglycans, heparan sulfate, and chondroitin, to attach to the cell surface (18–21). This is followed by internalization via fusion either at the plasma membrane (22, 23) or in acidified endosomes following macropinocytosis (24–26). The fusion of viral and host membranes via either route utilizes a complex membrane fusion apparatus made up of 11 to 12 viral proteins (25). Entry of VACV into a cell is still incompletely understood, with different strains of the virus utilizing different routes to enter different cell types (25, 27–30).

Once inside the cell, VACV initiates complex and largely ill-defined pathway manipulations to render the intracellular environment optimal for virus replication. For example, it possesses numerous mechanisms to evade the host immune response, with the inhibition of NF-κB activation being especially robust (31). VACV manipulates MAPK pathways during replication (32, 33), including stimulation of JNK in a sustained manner during the infectious cycle (13). In addition, VACV rapidly remodels the cytoskeleton of the cell, producing a distinctive cytopathic effect (CPE) characterized by cell rounding, loss of adhesion, and activation of cell migration within the first 2 to 3 h postinfection (p.i.), followed by cell flattening, reattachment, and projection formation (34–38). These virally induced cytoskeletal changes rely at least in part on inhibition of RhoA signaling pathways by the virally encoded F11 protein (39–41) and mirror alterations seen in wound healing and tumor metastasis (36), making VACV-host cell interactions relevant to a wide spectrum of biomedical research.

In order to elucidate how VACV manipulates host cell pathways, we investigated the role of TRAF2, a key pathway regulator, in VACV replication. We discovered that TRAF2 is a proviral factor in VACV replication in both HeLa cells and mouse embryonic fibroblasts (MEFs) and that its influence is exercised through promotion of efficient virus entry.

## MATERIALS AND METHODS

### Cells and viruses.

Cells (African green monkey kidney epithelioid [BS-C-1] cells, human cervix carcinoma epithelioid [HeLa] cells, and MEFs) were grown in Dulbecco's modified Eagle's medium (DMEM; Life Technologies) containing 50 IU/ml penicillin, 50 μg/ml streptomycin (Sigma), and 10% fetal bovine serum (FBS; Life Technologies). TRAF2^+/+^ and TRAF2^−/−^ MEFs (42) were sent by Søren R. Paludan (University of Aarhus, Aarhus, Denmark) with the permission of Tak W. Mak (Campbell Family Institute for Breast Cancer Research, Canada). Cells were incubated at 37°C in a 5% CO_2_ incubator. VACV strain Western Reserve (VACV) and VACV A5L-EGFP, in which enhanced green fluorescent protein (EGFP) is fused to the VACV core protein A5 (43), were kind gifts from Geoffrey L. Smith (University of Cambridge, Cambridge, United Kingdom). The experiments presented here were carried out with a sucrose gradient-purified IMV form of VACV or VACV A5L-EGFP.

### siRNA knockdown of TRAF2.

HeLa cells (4 × 10^3^ cells in 80 μl) were seeded in 96-well plates and incubated for 24 h at 37°C. Before transfection, media were removed and replaced with DMEM containing 5% FBS and no antibiotics. TRAF2 small interfering RNA (siRNA) SMARTpool and four deconvoluted (DC) TRAF2 siRNAs (Dharmacon/Thermo Scientific) were diluted to 0.3 μM in 1× siRNA buffer (Dharmacon/Thermo Scientific). The sequences of the DC siRNAs were as follows: TRAF2 DC1, GGAGCAUUGGCCUCAAGGA; TRAF2 DC2, GCAGGUACGGCUACAAGAU; TRAF2 DC3, CGGUAGAGGGUGAGAAACA; and TRAF2 DC4, GAAGAAGGCAUUUCUAUUU. Ten microliters of Dharmafect 1 transfection reagent (Dharmacon/Thermo Scientific) diluted in DMEM to a final concentration of 0.15% was added to the siRNAs and incubated for 20 min. Twenty microliters of this mixture was added to the cells, which were then incubated for 48 h at 37°C. After this period, media were removed from the plates by inversion and cells were infected with VACV A5L-EGFP at a multiplicity of infection (MOI) of 0.1 for 1 h at 37°C. Inocula were removed, and cells were overlaid with DMEM containing 2.5% FBS (2.5% DMEM). At different times p.i., fluorescence levels were measured using a Synergy HT MultiMode microplate reader (BioTek). Experiments were carried out in triplicate. Results were analyzed using a *t* test.

### qPCR confirmation of siRNA knockdown.

HeLa cells were seeded and transfected with TRAF2 siRNA SMARTpool or mock transfected as described above. After 48 h, samples from 24 wells for each sample were pooled and total RNA was extracted with the TRIzol reagent (Life Technologies), according to the manufacturer's instructions. cDNA was generated by using either an ImProm system (Promega) or a Pure Link RNA minikit (Life Technologies) and quantified by quantitative PCR (qPCR) using SYBR green PCR master mix (Applied Biosystems/Life Technologies) or a Rotor-Gene SYBR green reverse transcription-PCR kit (Qiagen) on a Rotor-Gene Q machine (Qiagen). Technical duplicates were performed for all samples. The relative expression of TRAF2 was calculated using the Pfaffl method (44) and normalized against GAPDH (glyceraldehyde-3-phosphate dehydrogenase) (45), hypoxanthine phosphoribosyltransferase (HPRT) (46), and beta-glucuronidase (GUSB) (47) mRNAs using geNorm analysis (48). Data were analyzed using a one-sample *t* test in the statistical package GenStat. Primers used for TRAF2 were forward primer 5′-CACCGGTACTGCTCCTTCTG-3′ and reverse primer 5′-TGAACACAGGCAGCACAGTT-3′.

### Single-step and multistep growth curves.

For single-step (or one-step) growth curves, TRAF2^+/+^ and TRAF2^−/−^ MEFs were infected with VACV at an MOI of 10 for 1 h at 37°C. The inoculum was removed (time point 0 h), and cells were washed with medium and incubated with 2.5% DMEM. At 0, 4, 8, 12, and 24 h p.i., supernatants were collected and centrifuged at low speed to remove cell debris. Supernatants were then incubated with Rb168 antibody (49) (a kind gift from Geoffrey L. Smith, University of Cambridge, Cambridge, United Kingdom) for 1 h at 37°C in order to neutralize IMV particles. The titers of the virus present in the supernatants were determined by plaque assay on BS-C-1 cells. Cells were scraped into medium, collected by centrifugation, and pooled with the cell debris that had been collected by centrifugation of the supernatant. These cells were frozen and thawed three times and sonicated, and the titers of the cell-associated fractions were determined by plaque assay on BS-C-1 cells. For the multistep growth curves, TRAF2^+/+^ and TRAF2^−/−^ MEFs were infected at an MOI of 0.01 for 1 h at 37°C. Cells were harvested at 0, 4, 8, 12, 24, and 48 h p.i. by scraping them into the medium, frozen and thawed three times, and sonicated. Virus titers were also determined by plaque assay on BS-C-1 cells. The multistep growth curve experiment was carried out three times, and the single-step growth curve experiment was carried out twice. Log transformations of the titration data were analyzed using a repeated-measures mixed model for the multistep growth curve and the cell-associated and supernatant components of the single-step growth curve. Each model fitted type time and the type-time interaction as fixed effects and replicate, replicate-type, replicate-time, and replicate-type-time as random effects. A different level of variability was assumed at each time point. The inclusion of random replicate effects and their interactions as random allows the variation in results between replicates to be taken into account in carrying out statistical tests and in calculating confidence intervals (50).

### Virus titration.

Serial dilutions of samples were made in 2.5% DMEM. Dilutions were inoculated in duplicate onto confluent monolayers of BS-C-1 cells in 6-well plates. After infection for 1 h at 37°C, the inocula were aspirated, cells were overlaid with 2 ml of 1.5% carboxy methylcellulose (Sigma) in 2.5% DMEM, and cells were incubated for 2 days at 37°C. The semisolid overlay was aspirated, and cells were washed briefly with phosphate-buffered saline (PBS) and stained with 0.1% (wt/vol) crystal violet (Sigma) in 15% ethanol. After rinsing with water, plates were air dried and the number of plaques was determined.

### Measurement of viral plaque size.

Semiconfluent monolayers of TRAF2^+/+^ and TRAF2^−/−^ MEFs were infected with VACV for 1 h at 37°C. The inoculum was removed (time point 0 h), cells were washed with medium, 2 ml of 1.5% carboxy methylcellulose (Sigma) in 2.5% DMEM was added, and cells were incubated for 2 days at 37°C. The semisolid overlay was then aspirated, and cells were washed briefly with PBS and stained with 0.1% (wt/vol) crystal violet (Sigma) in 15% ethanol. Images of individual plaques were taken using a Zeiss Axiovert 40 CFL inverted microscope and a Canon Powershot A640 camera. The diameter of each plaque was measured using Adobe Photoshop image software.

### Protein analysis.

TRAF2^+/+^ and TRAF2^−/−^ MEFs were infected with VACV or mock infected for 1 h at 37°C. After removal of the inocula, cells were overlaid with 2.5% DMEM and whole-cell lysates were collected into lysis buffer (51) at the times indicated below. When required, cells were pretreated with 50 nM bafilomycin A (Sigma) for 1 h before VACV infection (52).

### Wound healing.

Confluent monolayers of TRAF2^+/+^ and TRAF2^−/−^ MEFs in a 6-well plate were scored using a 200-μl pipette tip to generate wounds devoid of adherent cells. Monolayers were infected at an MOI of 5 for 1 h on ice with VACV A5L-EGFP and then incubated at 37°C. At the times indicated below, the monolayers were examined using a Zeiss Axiovert 40 CFL inverted microscope and the number of cells which had migrated into the wound in each well was counted. Representative images were captured using a Canon Powershot A640 camera.

### Electrophoresis and immunoblotting.

Whole-cell extracts were separated by electrophoresis on 12.5% SDS-polyacrylamide gels and transferred onto nitrocellulose membranes. The membranes were washed with PBS containing 0.1% Tween 20 and incubated with specific phospho-SAPK/JNK (Cell Signaling) and SAPK/JNK (Cell Signaling) antibodies. Immunoreactive bands were visualized using an Immun-Star WesternC chemiluminescence kit (Bio-Rad). Detection of TRAF2, p84, A46, and D8 was carried out using direct infrared fluorescence (Li-Cor). Blots were treated with Odyssey blocking buffer (Li-Cor) before incubation with A46 (53), D8 (54), TRAF2 (Santa Cruz), p84 (GeneTex), or β-actin (AbCam) antibodies. Goat anti-rabbit IgG (H+L) DyLight 800 conjugate (Cell Signaling) and goat anti-mouse IgG (H+L) DyLight 680 conjugate (Cell Signaling) were used as secondary antibodies. Blots were scanned in an Odyssey scanner, and bands were quantified using Odyssey scanning software. The Odyssey system uses infrared fluorescent signals, rather than enzymatic reactions, to detect protein bands on a membrane, making signal intensity linear within a large dynamic range and enabling accurate quantification of the amount of protein present (55, 56). A mixed model was used to analyze the effect of bafilomycin A treatment in the expression of the early viral protein A46 in TRAF2^+/+^ and TRAF2^−/−^ MEFs following VACV infection. In this model, we have fitted line, treatment, time, and treatment-line, line-time, treatment-time, and line-treatment-time interactions as fixed effects. The variation between experiments was taken into account by fitting experiment, treatment-experiment, time-experiment, and time-treatment-experiment as random effects (50).

### Flow cytometry.

TRAF2^+/+^ and TRAF2^−/−^ MEFs were infected with VACV A5L-EGFP (MOI = 10) for 1 h at 4°C. After the inocula were removed, cells were either processed immediately (0 h p.i.) or incubated at 37°C for 1 h. At 0 h, cells were either scraped into medium or trypsinized for 10 min at 37°C to remove bound virus. At 1 h p.i., cells were trypsinized for 10 min at 37°C. Cells were then washed in PBS, fixed in formalin for 30 min at 4°C, centrifuged at low speed, and resuspended in 500 μl of ice-cold PBS. When needed, cells were pretreated with 5 μM JNK inhibitor VIII (JNKi; Merck Millipore) (13) for 1 h prior to infection, incubated for 1 h p.i., and processed as described above. Mock-infected samples were included in parallel in every experiment. Samples were analyzed using a BD FACSCalibur flow cytometer and BD CellQuest software or the FlowJo software package.

### Confocal microscopy.

Uncoated VACV cores were labeled and visualized using an anticore rabbit polyclonal serum that recognizes the core proteins A10, A3, F18, L4, and A4 (57). This serum was kindly provided by Geoffrey L. Smith (University of Cambridge, Cambridge, United Kingdom). TRAF2^+/+^ and TRAF2^−/−^ MEFs were seeded at 10^5^ cells/ml in round coverslips in a 6-well plate and incubated for 24 h at 37°C. Cells were infected with VACV (MOI = 5) for 1 h at 4°C. Inocula were removed, and cells were overlaid with 2.5% DMEM. At different time points p.i., the medium was removed, and cells were gently washed three times with ice-cold PBS and then fixed with PBS containing 4% (wt/vol) paraformaldehyde for 20 min on ice and then for 40 min at room temperature (RT). Cells were then washed again three times with PBS and permeabilized with PBS containing 2% FBS and 0.1% (wt/vol) saponin (Sigma). The samples were then incubated for 1 h at RT with mouse monoclonal antibody directed against D8 (1:300) and rabbit polyclonal anticore antiserum (1:500). After three washes in PBS with 2% FBS, the samples were incubated for 1 h at RT with Alexa Fluor 488-conjugated antirabbit antibody and Alexa Fluor 594-conjugated antimouse antibody (Life Technologies). After three further washes in PBS, the samples were mounted with ProLong Gold antifade reagent with DAPI (4′,6-diamidino-2-phenylindole; Molecular Probes). Images were collected using a Zeiss LSM 710 confocal microscope and Zen 2011 software (Zeiss).

## RESULTS

### TRAF2 has a proviral role in VACV replication.

**FIG 1:**
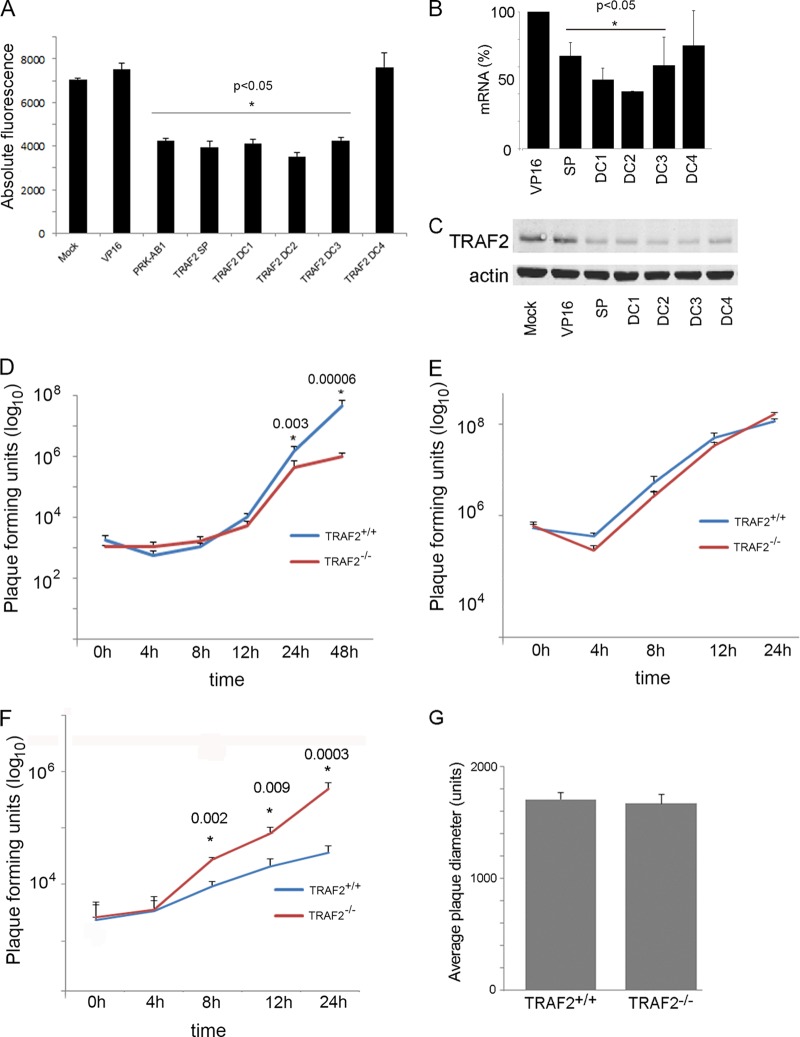
TRAF2 plays a role in VACV replication. (A) HeLa cells were transfected in triplicate with a TRAF2 siRNA SMARTpool (SP) and four individual deconvoluted TRAF2 siRNAs (DC1 to DC4). After 48 h, cells were infected with VACV A5L-EGFP at 0.1 PFU/cell, and at 48 h p.i., fluorescence levels were measured. The experiment was carried out three times in triplicate to produce a data set of three biological replicates each containing three technical replicates, and the results were analyzed using a *t* test. *P* values of <0.05 are noted. (B) HeLa cells were transfected with siRNA targeting VP16, TRAF2 SMARTpool, and TRAF2 DC1, DC2, DC3, and DC4. After 48 h, cells were harvested, RNA was extracted, and qPCR was performed to analyze the knockdown of TRAF2 by siRNAs. TRAF2 expression values in relation to those for the VP16-transfected samples are shown. Error bars indicate the standard errors of the means. *P* values (one-sample *t* test) of <0.05 are noted. (C) Western blot image of protein lysates from HeLa cells mock transfected or transfected with siRNA targeting VP16, TRAF2 SMARTpool, or TRAF2 DC1, DC2, DC3, or DC4, probed with antibody raised against TRAF2 (top) or β-actin (bottom), and visualized using direct infrared fluorescence (Li-Cor) in an Odyssey scanner. (D) Multistep growth curve. TRAF2^+/+^ and TRAF2^−/−^ MEFs were infected with VACV at 0.01 PFU/cell. Virus was harvested at the indicated times p.i. Total virus levels were determined by plaque assay on BS-C-1 cells. The graph shows the mean titer of three biological replicates. Error bars indicate the standard errors of the means. *P* values of <0.05 are noted. (E and F) Single-step growth curve. TRAF2^+/+^ and TRAF2^−/−^ MEFs were infected with VACV at 10 PFU/cell. At the indicated times p.i., cells (E) and supernatant (F) were collected and virus titers were determined by plaque assay on BS-C-1 cells. The graphs show the mean titers of two biological replicates. Error bars indicate the standard errors of the means. (G) The average diameter of VACV plaques on TRAF2^+/+^ (*n* = 41) and TRAF2^−/−^ (*n* = 39) monolayers. Error bars indicate the standard errors of the means.

In order to examine the effect of TRAF2 on VACV replication, we reduced expression of the protein in HeLa cells using a SMARTpool of four different siRNAs targeting the mRNA. siRNA transfection was followed 48 h later by infection at a low multiplicity (MOI = 0.1) with VACV A5L-EGFP, in which enhanced green fluorescent protein (EGFP) is fused to the VACV core protein A5 (43), thus correlating fluorescence with VACV multiplication. EGFP fluorescence was quantified at 48 h p.i., allowing multiple viral replication cycles to be completed. These data were compared to those for mock-transfected cells, cells transfected with a nonspecific siRNA (targeting VP16 from herpes simplex virus type 1), and a positive-control siRNA which is known to inhibit VACV replication (targeting PRK-AB1). All wells were examined microscopically for cell death due to potential toxic effects of the siRNA; none was noted. The two negative controls (mock-transfected cells and cells transfected with a nonspecific siRNA targeting VP16) resulted in similar levels of fluorescence, while the siRNA targeting PRK-AB1 significantly reduced the levels of fluorescence (Fig. 1A). Knockdown of TRAF2 using the SMARTpool siRNA reduced fluorescence by approximately 40%, indicating that TRAF2 has a positive role in VACV replication (Fig. 1A). In this system, a reduction in fluorescence of 40% is equivalent to a reduction of approximately 1 log_10_ PFU (data not shown).

To rule out potential off-target effects of the siRNAs, the SMARTpool was deconvoluted; that is, the effects of the four TRAF2 siRNAs that made up the SMARTpool were tested individually. Transfection with three (DC1, DC2, and DC3) of the four deconvoluted individual siRNAs significantly reduced the fluorescence to a level similar to that of the SMARTpool (Fig. 1A), demonstrating the proviral role of TRAF2 in VACV replication. Transfection of the fourth siRNA (DC4) had no effect on VACV replication, suggesting that this particular siRNA is nonfunctional.

To assess the effect of the siRNA SMARTpool on TRAF2 mRNA levels, HeLa cells were transfected with the SMARTpool targeting TRAF2 and with the deconvoluted siRNAs, and after 48 h, RNA was extracted and the levels of TRAF2 mRNA were determined by qPCR and normalized to the levels of three housekeeping gene transcripts (GAPDH, HPRT, and GUSB). TRAF2 mRNA was statistically significantly knocked down by SMARTpool, DC1, DC2, and DC3, but not DC4 (Fig. 1B). In order to verify the effect of the siRNAs on TRAF2 protein levels, whole-cell lysates were collected and analyzed by immunoblotting at 48 h posttransfection. Expression of TRAF2 was reduced following transfection with TRAF2 SMARTpool and individual siRNAs (Fig. 1C). Thus, the knockdown of TRAF2 expression by siRNA was evident at both the mRNA and protein levels. Importantly, the DC4 siRNA was the least effective at reducing both TRAF2 mRNA and protein levels, correlating with its poor efficacy against virus replication (Fig. 1A).

To corroborate the siRNA results, we examined the kinetics of virus replication in MEFs derived from wild-type (WT) and TRAF2^−/−^ mice (42). The TNF-α-induced NF-κB pathway remained functional in TRAF2^−/−^ MEFs due to redundancy between TRAF2 and TRAF5 (58); however, these cells can be used to investigate the role of TRAF2 in other signaling pathways. TRAF2^+/+^ and TRAF2^−/−^ cells were infected with VACV at low (multistep) and high (single-step) MOIs, samples were harvested at different times p.i., and virus titers were determined by plaque assay. The multistep growth curve experiment was carried out three times, and the single-step growth curve experiment was carried out twice. Data from the replicates were highly reproducible and in both cases were analyzed for statistical significance using a mixed model (50) that incorporated data from all replicates.

In the multistep growth curve experiment, VACV replicated to a significantly lower titer in cells that lacked TRAF2 (*P* < 0.01 at 24 h and 48 h p.i.; Fig. 1D). At 48 h p.i., the difference was 1.73 log_10_ PFU. This confirmed our previous results showing that TRAF2 is important for VACV replication. In a single-step growth curve experiment, there was no significant difference in viral titers in the cell-associated fraction (Fig. 1E); however, there was a significant increase in the amount of EEV present in the supernatant of TRAF2^−/−^ cells at 8, 12, and 24 h p.i. (Fig. 1F). This finding was unexpected, given the reduction in virus growth in the absence of TRAF2 under multicycle replication conditions. However, VACV Western Reserve produces less than 1% of its progeny virions as EEV (59); therefore, the increase in EEV production in the TRAF2^−/−^ cells is most likely masked in the multistep growth curve experiment by the overwhelming numbers of IMVs produced by the infected cells.

Viral plaque size has been shown to correlate with production of actin tails (discussed in reference 16). To determine whether TRAF2 influenced this aspect of VACV replication, confluent monolayers of TRAF2^+/+^ and TRAF2^−/−^ MEFs were infected at a low MOI (approximately 20 PFU per well) to allow individual plaques to form over 48 h. The plaques were then visualized with crystal violet, and the relative diameter was measured. No significant difference (*P* > 0.05) between VACV plaques on TRAF2^+/+^ and TRAF2^−/−^ MEFs was detected (Fig. 1G).

Overall, these results show that the loss of normal TRAF2 expression significantly reduces the titer of VACV replication under multicycle conditions, identifying it as a proviral factor.

### Loss of TRAF2 delays the production of early and late VACV proteins.

**FIG 2:**
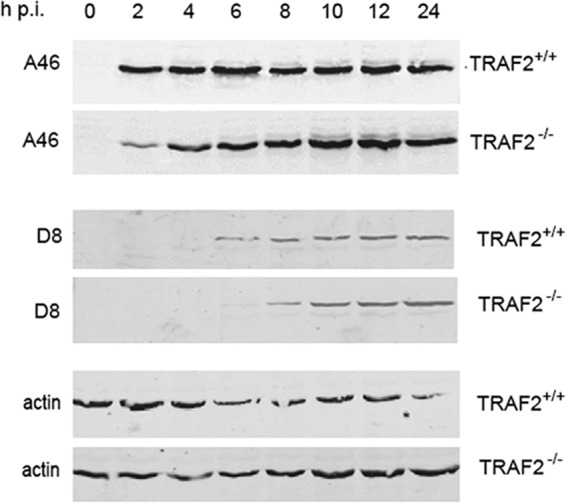
Loss of TRAF2 delays the expression of early and late VACV proteins. TRAF2^+/+^ and TRAF2^−/−^ MEFs were infected with VACV at 5 PFU/cell or mock infected for 1 h at 37°C. Unbound virus was removed by washing with PBS, and cells were incubated with 2.5% DMEM. Whole-cell lysates were collected at 0, 2, 4, 6, 8, 10, 12, and 24 h p.i. Proteins were separated by SDS-PAGE and analyzed by immunoblotting with the indicated antibodies, using direct infrared fluorescence (Li-Cor) in an Odyssey scanner.

To identify the stage at which a deficiency of TRAF2 hinders VACV replication, we examined the timing of production of early and late VACV proteins in TRAF2^+/+^ and TRAF2^−/−^ MEFs by Western blotting of cell lysates at various times following infection at a high multiplicity (MOI = 10). Equal amounts of protein were loaded in each well. The presence of equal amounts was confirmed by blotting with an antibody which recognizes β-actin, which served as a loading control. Expression of a viral early protein, A46 (53), was first detected at 2 h p.i. in both TRAF2^+/+^ and TRAF2^−/−^ cells, but the amount present in the absence of TRAF2 was markedly reduced (Fig. 2). By 6 h p.i., the difference in the amount of A46 was much less evident. Detectable expression of a representative late protein, D8 (54), was similarly delayed in TRAF2^−/−^ MEFs from 6 to 8 h p.i., but again, it recovered to normal levels by 10 h p.i. (Fig. 2). These results indicate that loss of TRAF2 delays VACV replication rather than prevents it, as a consequence of TRAF2 facilitating an early stage of the VACV life cycle.

### TRAF2 is required for VACV-mediated activation of JNK.

**FIG 3:**
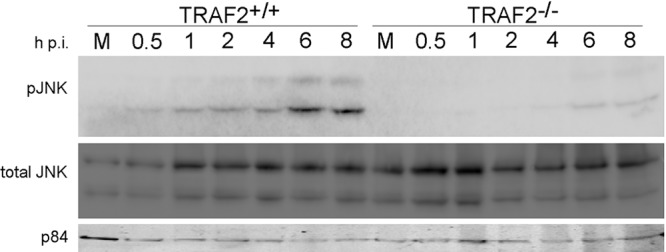
TRAF2 is required for activation of JNK following VACV infection. TRAF2^+/+^ and TRAF2^−/−^ MEFs were infected with VACV at 10 PFU/cell or mock infected. Whole-cell lysates were collected at the times indicated. Proteins were separated by SDS-PAGE and analyzed by immunoblotting with the indicated antibodies. M, mock-infected cells; pJNK, phosphorylated JNK.

VACV infection activates the JNK pathway early in infection (by 3 h p.i.) (13), and TRAF2 is known to activate JNK downstream of TNFR1 (7). Further, a rise in EEV release from infected cells but no difference in the amount of cell-associated virus has been described in a one-step growth curve experiment of VACV replication in JNK1/2^−/−^ cells (13), findings very similar to our findings (described above) in TRAF2^−/−^ MEFs. We therefore examined whether VACV-induced phosphorylation of JNK occurs via TRAF2. TRAF2^+/+^ and TRAF2^−/−^ MEFs were infected with VACV, and whole-cell extracts were prepared. Immunoblotting analysis was carried out for phosphorylated JNK, total JNK, and p84 as an additional loading control (Fig. 3). This revealed that while the levels of total JNK were not altered by VACV infection (Fig. 3, middle), JNK phosphorylation in response to VACV infection was considerably reduced in the absence of TRAF2 (Fig. 3, top). In TRAF2^+/+^ cells, increased JNK phosphorylation was detectable as soon as 0.5 h p.i., with phosphorylation increasing up to 6 h p.i. In TRAF2^−/−^ cells, phosphorylation was first detected very faintly at 4 h p.i. and more strongly at 6 and 8 h p.i. This indicates that TRAF2 is required for early VACV-mediated activation of JNK, but by 4 h p.i., some TRAF2-independent VACV-stimulated activation of JNK occurs.

### The absence of TRAF2 alters cytoskeletal responses to VACV infection.

**FIG 4:**
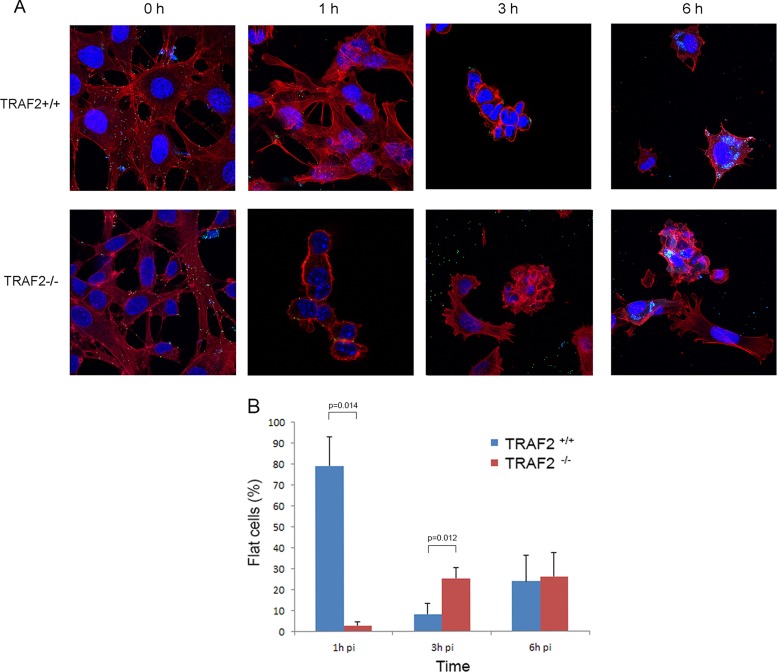
Altered cytopathic effect in TRAF2^−/−^ MEFs following VACV infection. (A) TRAF2^+/+^ and TRAF2^−/−^ MEFs were infected with VACV A5L-EGFP at 5 PFU/cell for 1 h at 4°C and then fixed at the indicated times p.i. before being labeled with Texas Red-phalloidin (red) and DAPI (blue). Images were collected with a Zeiss LSM 710 confocal microscope. (B) Cells with a flat and round morphotype were counted at the indicated times p.i., and the results were analyzed using a *t* test. Data are from three independent experiments. *P* values of <0.05 are noted.

While performing the growth curve experiments, we noticed that the morphology of TRAF2^+/+^ and TRAF2^−/−^ MEFs varied after VACV infection. In order to compare the CPE caused by VACV, cells were infected with VACV A5L-EGFP at an MOI of 5 on ice to allow virus to attach to but not enter cells. At 0 h p.i., the inoculum was removed and the cells were examined. Minimal CPE was noted in either cell line at this time point (Fig. 4A). The cultures were then placed at 37°C in 5% CO_2_ and reexamined at regular intervals. At 1 h p.i., most TRAF2^+/+^ cells (∼80%) were still flat, with a minority of cells being shrunken and rounded (Fig. 4A and B). As the infection progressed, the TRAF2^+/+^ cells continued to contract, as expected in VACV-infected cells, with almost all cells (>90%) being rounded up at 3 h p.i. At 6 h p.i., 24% of the cells had assumed a flattened, angular morphology, with some short projections being visible. This sequence of initially rounding up and then flattening out and producing projections has been reported previously in a variety of cell types in response to VACV infection (35, 37). We found that the loss of TRAF2 led to marked differences in response to VACV infection. Soon after infection, TRAF2^−/−^ MEFs showed rapid and pronounced cell contractility, with almost all cells (97%) being rounded after only 1 h (Fig. 4A and B). After the initial and rapid contraction of TRAF2^−/−^ MEFs, the cells gradually reverted to the angular, flattened morphotype. At 3 h p.i., the changes in the TRAF2^−/−^ cells still proceeded those in the TRAF2^+/+^ cells, with significantly more flat TRAF2^−/−^ cells being detected. At 6 h p.i., the influence of TRAF2 on the cytopathic effect had ceased, with similar numbers of flattened and rounded cells being detected in infected TRAF2^+/+^ and TRAF2^−/−^ MEF monolayers (Fig. 4A and B).

**FIG 5:**
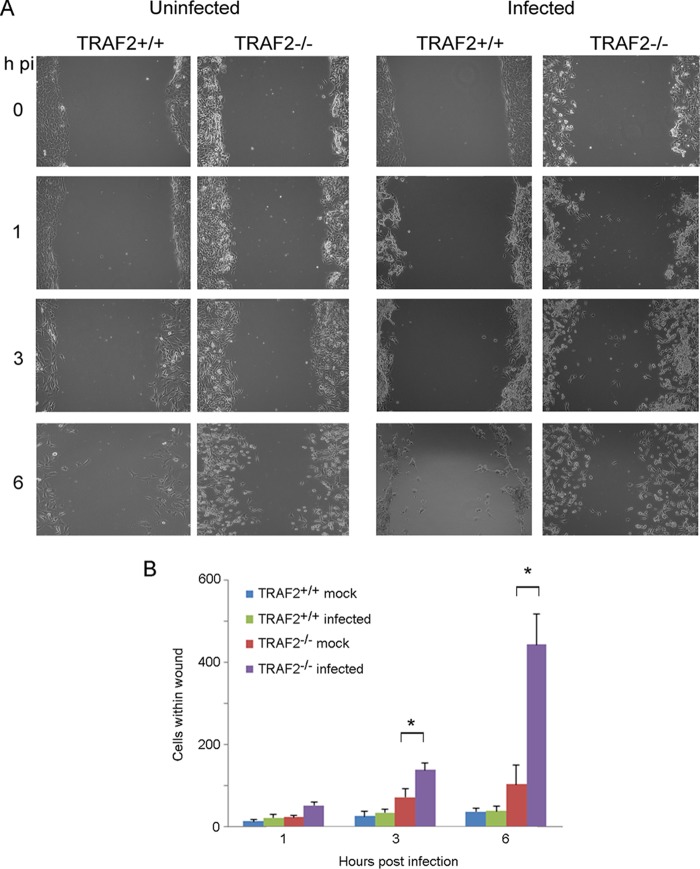
Loss of TRAF2 increases VACV-induced cell motility. TRAF2^+/+^ and TRAF2^−/−^ MEFs were scored using a 200-μl pipette tip, before being mock infected or infected with VACV A5L-EGFP at 5 PFU/cell for 1 h on ice and then incubated at 37°C. (A) The monolayers were examined at the indicated times; representative images are shown. (B) The number of cells which had migrated into the wound in each well was counted, and the results were analyzed using a *t* test. Data are from three independent experiments. *, *P* < 0.05.

The rounding up of VACV-infected cells is associated with a loss of adhesion and increase in cell motility (35, 36, 41). Since rapid rounding up is seen in TRAF2^−/−^ MEFs infected with VACV, we used the *in vitro* wound healing experiment to determine whether this was associated with an increase in cell motility. Confluent monolayers of TRAF2^+/+^ and TRAF2^−/−^ MEFs were scratched to create a wound and then infected with VACV at an MOI of 5 or mock infected. At 1, 3, and 6 h p.i., images of the wound were taken and the number of cells which had migrated into the cell-free region was counted (Fig. 5A and B). In uninfected cells, a greater number of TRAF2^−/−^ cells than TRAF2^+/+^ MEFs migrated into the wound, suggesting that cells lacking TRAF2 have an increased propensity to migrate. This increased motility was magnified when the cells were infected with VACV, when by 6 h p.i., 10-fold more VACV-infected TRAF2^−/−^ MEFs than TRAF2^+/+^ MEFs had migrated into the wound. Thus, overall these results suggest that TRAF2 acts to dampen down VACV-induced cytoskeletal rearrangements early postinfection.

### The absence of TRAF2 results in reduced numbers of uncoated viral particles within the cytoplasm.

**FIG 6:**
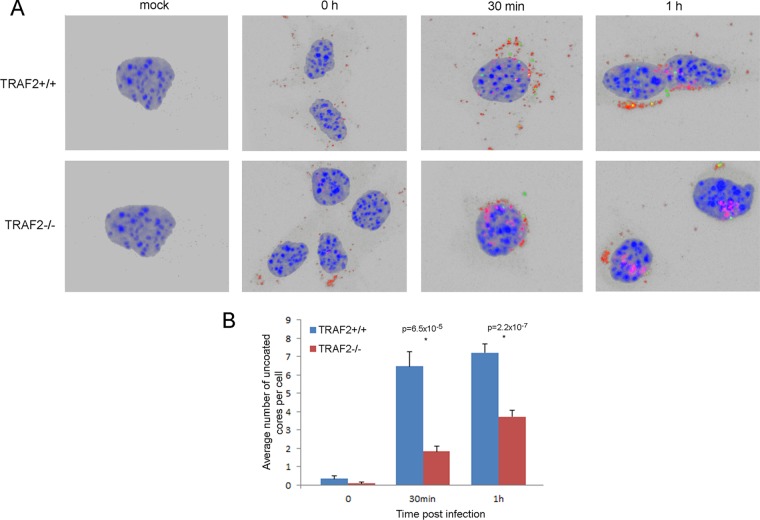
VACV entry is delayed in MEFs lacking TRAF2. (A) TRAF2^+/+^ MEFs (top) and TRAF2^−/−^ MEFs (bottom) were mock infected or infected with VACV at 10 PFU/cell for 1 h at 4°C. The cells were then fixed after 0 h, 0.5 h, or 1 h of incubation at 37°C. Mock-infected cells were incubated for 1 h. Cells were then labeled with anticore antibody, which labels uncoated virus cores (green), or anti-D8 antibody (red) and the nuclear stain DAPI (blue). z-stack images of at least 10 randomly selected cells per treatment group were collected and assembled using 3D reconstruction software (Zen Black, 2011), and the number of green core particles per cell was counted. A representative image of each sample is shown (3D reconstruction, maximum-intensity projection mode). (B) The average number of uncoated cores in TRAF2^+/+^ and TRAF2^−/−^ MEFs at the times indicated. Error bars represent standard errors of the means. Results were analyzed using a *t* test. *P* values of <0.05 are shown.

The delay in VACV protein expression observed in the TRAF2-knockout cells could be explained by a defect in virus entry. To examine this hypothesis, we employed a virus particle uncoating assay based on confocal microscopy of cells stained with an antibody directed against the VACV core, whose epitope is not accessible unless uncoating has occurred (57). TRAF2^+/+^ and TRAF2^−/−^ MEFs were infected or mock infected with VACV on ice for 1 h at an MOI of 5 and then either fixed immediately or fixed after incubation at 37°C for various periods of time. Samples were then labeled with antibodies directed against D8, which is present on the IMV membrane, and anticore antibody, which detects only uncoated VACV cores and not IMV or EEV particles (57). This method is regarded as the most sensitive and specific for studying VACV entry (30). The entire volume of randomly selected cells (at least 10 per treatment group) was imaged by capturing serial confocal z-sections. The z-stacks were then assembled and examined using a three-dimensional (3D) reconstruction view to determine the number of uncoated cores per cell (Fig. 6A). No D8 or anticore antibody labeling was detected in mock-infected controls or in infected cells in which the primary antibodies were omitted from the procedure (data not shown). At 0 h p.i., D8 staining (red) was seen on the surface of infected TRAF2^+/+^ and TRAF2^−/−^ cells, indicating that virions had attached, but uncoated cores (green labeling) were rare. Uncoated cores could be identified at both 30 min and 1 h p.i. in TRAF2^−/−^ and TRAF2^+/+^ MEFs as punctate green structures within the cytoplasm. These were counted and compared (Fig. 6B), revealing that there were statistically significantly fewer cores in TRAF2^−/−^ MEFs than TRAF2^+/+^ MEFs and, thus, that TRAF2 promotes VACV entry. In the TRAF2^+/+^ MEFs, the majority of the virions appeared to enter and uncoat within 30 min of infection, with only a small increase in the number of uncoated cores per cell being detected at between 30 min and 1 h. In contrast, the number of uncoated cores in the TRAF2^−/−^ MEFs continued to increase between 30 min and 1 h p.i., suggesting that the loss of TRAF2 delays, rather than prevents, entry.

### The TRAF2-associated delay in VACV entry is not mediated by the JNK pathway.

**FIG 7:**
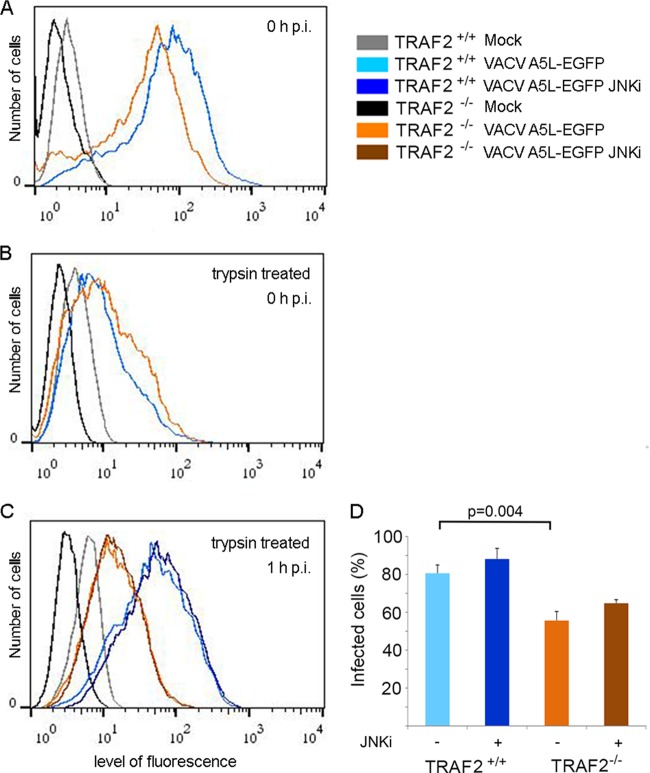
Delayed VACV entry in MEFs is JNK independent. (A) TRAF2^+/+^ and TRAF2^−/−^ MEFs were infected with VACV A5L-EGFP at 10 PFU/cell for 1 h at 4°C. Cells were scraped into medium at 0 h p.i. and fixed, and fluorescence was quantified by flow cytometry. (B) TRAF2^+/+^ and TRAF2^−/−^ MEFs were infected with VACV A5L-EGFP at 10 PFU/cell for 1 h at 4°C. Cells were trypsinized for 10 min at 0 h p.i., fixed, and processed as described above. (C) TRAF2^+/+^ and TRAF2^−/−^ MEFs were infected with VACV A5L-EGFP at 10 PFU/cell for 1 h at 4°C. Cells were collected at 1 h p.i., trypsinized for 10 min, and processed as described above. Additional samples were pretreated with 5 μM JNKi for 1 h prior to infection, incubated for 1 h p.i., and then processed. (D) The average number of infected cells at 1 h p.i. calculated from flow cytometry data from five independent experiments. Error bars represent standard errors of the means. *P* values of <0.05 (*t* test) are shown.

Slower VACV entry into the TRAF2-knockout cells could result from either reduced virus binding to the cell surface or an alteration in the subsequent entry process. To test between these possibilities, we analyzed VACV A5-EGFP binding to TRAF2^+/+^ and TRAF2^−/−^ MEFs by flow cytometry (29, 60). Cells were infected (or mock infected) on ice for 1 h, washed three times with ice-cold PBS, and then either immediately fixed to examine viral attachment (Fig. 7A) or treated with trypsin to detach surface-bound virions before being fixed (Fig. 7B and C). In all cases, only background levels of fluorescence were seen from mock-infected cells, while, as expected, substantial signals were obtained in the presence of fluorescently labeled virus. When the samples were examined at 0 h p.i., cell-associated fluorescence in the absence of trypsin treatment was very similar in the TRAF2^+/+^ and TRAF2^−/−^ cells (Fig. 7A) and was similarly reduced by trypsin treatment, as seen by the shift in the fluorescence curves of both the TRAF2^+/+^ and TRAF2^−/−^ infected cells to the left in Fig. 7B. Thus, the presence or absence of TRAF2 did not affect attachment of virions to the surface of the cell. However, if the cells were incubated at 37°C for 1 h to allow entry to proceed prior to treatment with trypsin and fixation, the majority of the viral fluorescence was trypsin resistant in the TRAF2^+/+^ cells, but a substantial proportion of the signal was still trypsin sensitive in the TRAF2^−/−^ cells (Fig. 7C). The fluorescence profiles of mock-infected cells were used to differentiate between infected and uninfected cells, and the results were quantified (Fig. 7D). At 1 h p.i., significantly more TRAF2^+/+^ MEFs than TRAF2^−/−^ MEFs contained internalized virions. Thus, TRAF2 has a role in facilitating VACV entry into MEFs but does not affect virus attachment.

To test if the decreased ability of VACV to activate the JNK signaling pathway in the absence of TRAF2 was involved in the entry defect, we used a JNK inhibitor (JNKi [13]) to see if this would reproduce the effect of the loss of TRAF2 in MEFs. However, pretreatment of TRAF2^+/+^ MEFs (or TRAF2^−/−^ MEFs) with JNKi did not reduce virus entry (Fig. 7C and D), indicating that TRAF2 influences VACV entry in a JNK-independent manner.

### TRAF2 may act in the plasma membrane-associated entry pathway.

**FIG 8:**
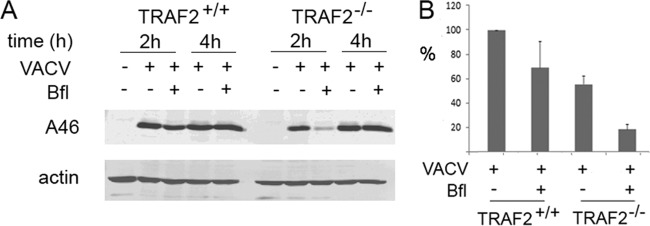
VACV entry in TRAF2^−/−^ cells is markedly reduced by bafilomycin A treatment. (A) TRAF2^+/+^ and TRAF2^−/−^ MEFs were infected with VACV at 1 to 10 PFU/cell for 1 h at 37°C. Some cells were pretreated with 50 nM bafilomycin A (Bfl) for 1 h before infection, with subsequent virus infection and overlay carried out in the presence of the chemical. Whole-cell lysates were collected at the indicated times p.i. Proteins were separated by SDS-PAGE and analyzed by immunoblotting with the indicated antibodies. (B) Proteins were quantified and normalized against the amount of β-actin using Odyssey scanning software. The data represented are the averages and standard errors of the means (error bars) from four biological replicates. Using mixed-model analysis, bafilomycin A treatment significantly influenced the expression of A46 in both TRAF2^+/+^ cells (*P* = 0.036) and TRAF2^−/−^ cells (*P* = 0.019).

VACV enters cells via one of two pathways, either fusion at the plasma membrane or endocytosis. To test which entry pathway is dependent on TRAF2, we treated cells with bafilomycin A, which blocks acidification of endosomes and thus prevents VACV entry by endocytosis (52). We pretreated TRAF2^+/+^ and TRAF2^−/−^ MEFs with bafilomycin A, infected the cells with VACV, and used early gene (A46) expression as a measure of viral entry. Similar to our previous results (Fig. 2), we found considerably less A46 protein (an approximately 50% reduction) in TRAF2^−/−^ MEFs than in TRAF2^+/+^ MEFs at 2 h p.i. (Fig. 8A). As expected, bafilomycin A treatment significantly reduced the expression of A46 in both TRAF2^+/+^ (*P* = 0.036) and TRAF2^−/−^ (*P* = 0.019) cells. In TRAF2^+/+^ MEFs, bafilomycin A treatment reduced the amount of A46 present at 2 h p.i. by 31% compared to the amount present in untreated TRAF2^+/+^ MEFs (Fig. 8A and B). This finding is similar to previous reports (30, 61) and consistent with the loss of the endosomal pathway and the increased dependence of the virus on plasma membrane fusion for entry. In comparison, bafilomycin A treatment of TRAF2^−/−^ MEFs had a much greater effect on virus entry, consistently reducing A46 expression by an average of 67% in treated versus untreated TRAF2^−/−^ MEFs at 2 h p.i. (Fig. 8A and B). These results reveal that VACV relies on the endosomal pathway to a greater extent in the absence of TRAF2 and therefore suggest that TRAF2 may facilitate entry of VACV via plasma membrane fusion rather than endocytosis.

## DISCUSSION

TRAF2 is a member of the TNF receptor-associated factor family. It is recruited to TNFR1 to effect activation of the canonical NF-κB pathway (62), the JNK pathway (63), and the p38 pathway (64), as well as inhibition of apoptosis (4). Less well defined roles for TRAF2 in the regulation of autophagy (65) and the epidermal growth factor receptor pathway (66) have been reported. VACV is known to modulate some of these TRAF2-regulated pathways; therefore, we investigated the role of TRAF2 in VACV replication. We found that siRNA knockdown of TRAF2 in HeLa cells hindered the replication of VACV (Fig. 1A). This result was confirmed in a multistep growth curve experiment in TRAF2^−/−^ MEFs (Fig. 1D). This VACV growth defect was not apparent in the cell-associated fraction of a one-step growth curve in TRAF2^−/−^ MEFs (Fig. 1E), indicating that the defect was not severe enough to be distinguished by plaque assay in a single replication cycle but required multiple rounds of replication to magnify the effect before it could be detected.

While no difference in the cell-associated fraction of the one-step growth curve was detected, the supernatant of infected TRAF2^−/−^ MEFs unexpectedly showed greater numbers of EEVs. Increased EEV production is an unusual finding which has previously been reported in viruses with a deleted or mutated A34R gene, such as VACV strain IHD-J (59, 67–69), or in cells lacking JNK1/2 (13). As TRAF2 has previously been reported to be involved in JNK activation, we examined the role of TRAF2 in VACV-induced phosphorylation of JNK. TRAF2 was indeed found to be required for rapid JNK pathway activation, substantiating the previous report describing a correlation between VACV-induced JNK activation and EEV release (13). Moreover, our findings identify the activation of the JNK pathway by VACV to be at the level of the adaptor complex or higher since TRAF2 is a crucial component of the initial stages of activation of the JNK pathway. The mechanism by which JNK activation promotes EEV release is unknown but has been suggested to be linked to altered microtubule and actin organization in the absence of JNK activation, promoting transport of cell-associated enveloped virus (CEV) to the cell surface and release of EEV (13).

As described above, the increase in EEV production identified in a one-step growth curve did not translate into an increase in viral replication in a multistep growth curve. This may be because less than 1% of VACV Western Reserve virions form EEVs (59), and their impact is therefore easily masked by overwhelming numbers of IMVs. Alternative explanations for the phenomenon are that increased numbers of EEVs produced from TRAF2^−/−^ cells are unable to reinfect TRAF2^−/−^ cells efficiently or that loss of TRAF2 results in a reduction in the production of CEVs. We investigated both of these scenarios and found that EEVs produced from TRAF2^−/−^ cells infected TRAF2^+/+^ and TRAF2^−/−^ cells equally well and that after 8 h of infection with VACV similar numbers of CEVs were present on the surface of TRAF2^+/+^ and TRAF2^−/−^ cells (data not shown). The effect of an increase in EEV production on the spread of VACV through a cell monolayer has not previously been investigated in detail; however, work with the mutant strain IHD-J suggested that increasing EEV levels have only a minor effect on overall spread through a monolayer (67), supporting the results from the TRAF2^−/−^ MEFs presented here.

**FIG 9:**
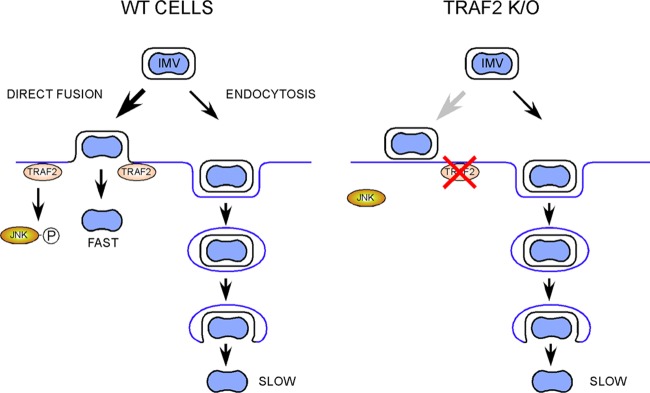
Proposed model for the role of TRAF2 in VACV entry. In normal cells, virus entry proceeds via either of two routes, direct entry at the plasma membrane or following endocytosis, with the former route forming the relatively fast route. In the absence of TRAF2, entry at the plasma membrane is inhibited and infection occurs predominantly via the relatively slow endocytic route. The absence of TRAF2 also inhibits early activation of JNK, but this is not relevant to virus entry.

As we detected an overall reduction in VACV replication in a multicycle growth curve in cells lacking TRAF2, we suspected that TRAF2 played an additional role, other than JNK activation, in the VACV life cycle and that this role was proviral. In order to uncover this proviral role of TRAF2 in VACV replication, we examined viral protein expression during a single-step growth curve experiment comparing TRAF2^+/+^ and TRAF2^−/−^ MEFs. This revealed that despite no overall difference in viral growth in the cell-associated fraction, there was a delay in both early and late protein expression (Fig. 2), indicating a role for TRAF2 during early stages of viral replication. We measured virus entry using uncoating experiments (Fig. 6) and cell fluorescence (Fig. 7) and found that TRAF2 promotes VACV entry. Treatment of cells with a JNK inhibitor did not influence VACV entry (Fig. 7C), indicating that TRAF2 facilitates VACV entry independently of its role in the JNK activation pathway. Loss of TRAF2 did not result in an absolute failure of virus entry, since uncoated virions were still seen within TRAF2^−/−^ cells (Fig. 6A) and viral protein expression was delayed rather than prevented (Fig. 2 and 8). One explanation for the slower viral entry was that loss of TRAF2 impacted only one of the two main routes of VACV entry, either plasma membrane fusion or endocytosis. Bafilomycin A treatment was used to inhibit the endosomal pathway, and while this produced only a moderate reduction in virus entry in TRAF2^+/+^ cells, a much greater reduction was seen in TRAF2^−/−^ MEFs, revealing the overdependence of VACV on the endosomal route of entry in the absence of TRAF2 (Fig. 8A and B). This implies that the plasma membrane route of entry is not functioning normally in cells lacking TRAF2 and suggests that TRAF2 may assist VACV entry specifically via plasma membrane fusion. This hypothesis is attractive, since the endosomal pathway of VACV entry has been suggested to be slower than the plasma membrane pathway of entry (30), which would explain why the TRAF2^−/−^ defect results in a delay, rather than a block, in virus entry and, thus, why multiple rounds of replication are required for detection of the defect. This model is outlined in Fig. 9. The identification of a protein involved in plasma membrane entry would be noteworthy, since most recently identified cellular proteins with an influence on VACV entry have been shown to influence the endocytic pathway, such as VPEF (70), integrin β1 (71), and CD98 (72). Whether TRAF2 acts directly on the virion at the plasma membrane level or influences a specific pathway utilized by the virus during plasma membrane fusion remains to be established.

Our studies of the TRAF2^−/−^ MEFs also revealed a role for TRAF2 in the VACV-induced CPE. The initial cytoskeletal changes induced by VACV in the TRAF2^−/−^ MEFs were more rapid and extensive than those identified in the WT MEFs (Fig. 4), and cell motility was enhanced (Fig. 5), indicating that TRAF2 acts to curb the early CPE and cell motility induced by VACV infection. Changes to cell shape and motility in response to VACV infection depend on early viral gene expression (36). However, TRAF2-knockout cells display delayed virus entry and early gene expression but more rapid changes to cell shape and motility. This apparent contradiction may be due to the delayed early gene expression still being sufficient to trigger cytoskeletal alterations. The cytoskeletal changes induced by VACV are biologically important because they resemble those found in tumors and wound healing (36). The mechanisms by which VACV alters the cytoskeleton remain to be fully characterized, but the viral protein F11 plays a central role. F11 acts as a dominant negative protein in the RhoA-mDia pathway, preventing RhoA from interacting with ROCK and therefore suppressing pathway signaling. Early cell contraction, detachment, and motility are F11-dependent phenomena (39–41), and the protein is also involved in late projection formation and EEV release (39, 40, 73). F11 restores motility and induces a cytopathic effect in modified vaccinia virus Ankara and myxoma virus, both of which lack a functional F11 (38, 40, 74). One explanation of the exaggerated CPE observed in infected TRAF2^−/−^ MEFs is that TRAF2 is involved in RhoA stimulation. The absence of TRAF2 could lead to reduced RhoA stimulation, potentiating the effect of F11 and leading to more rapid and extensive cytoskeletal changes. However, while TNF-α activation has been linked to Cdc42, Rac, and RhoA activation (75–80), there is no evidence connecting TRAF2 to these pathways. In fact, two studies have shown that TRAF2 is not required for TNF-α-associated cytoskeletal manipulation (75, 79). An alternative explanation for the cytoskeletal changes seen in VACV-infected TRAF2^−/−^ MEFs is a lack of JNK1/2 phosphorylation. The JNK pathway is known to influence the VACV CPE at 3 and 6 h p.i. (13), and stimulation of the JNK pathway by the compound *trans*-2-hexadecenal results in cytoskeletal changes similar to those described in TRAF2^−/−^ MEFs infected with VACV (81). It would be interesting to compare the CPE seen in TRAF2^−/−^ and JNK1/2^−/−^ MEFs at 1 h after infection with VACV to see if similar changes are noted. However, comparison of results from cell motility experiments in VACV-infected TRAF2^−/−^ MEFs (this work) and JNK1/2^−/−^ MEFs (13) reveals opposing effects, with the loss of JNK1/2 reducing cell motility but the loss of TRAF2 enhancing motility, suggesting that the situation is not straightforward. The role of TRAF2 in regulating cytoskeletal responses, such as cell shape and motility, in the face of a VACV infection likely involves more pathways than the JNK pathway alone.

We have found that TRAF2 affects the VACV life cycle in three distinct manners: activating the JNK pathway, influencing cytoskeletal rearrangement, and promoting virus entry. The exact relationship between these three functions remains to be defined. To the best of our knowledge, this is the first report of TRAF2 being involved in virus entry. Further investigations into the mechanism of action of TRAF2 in VACV replication may shed new light on the function of this protein and provide novel regulatory tools for key cellular pathways.
